# Retinal regeneration by transplantation of retinal tissue derived from human embryonic or induced pluripotent stem cells

**DOI:** 10.1186/s41232-016-0004-7

**Published:** 2016-04-25

**Authors:** Hiroshi Shirai, Michiko Mandai

**Affiliations:** 1 Laboratory for Retinal Regeneration, RIKEN Center for Developmental Biology, 2-2-3 Minatojima-minamimachi, Chuo, Kobe, 650-0047 Japan; 2 Department of Ophthalmology, Nagoya University Graduate School of Medicine, 65 Tsuruma-cho, Showa, Nagoya, 466-8550 Aichi Japan

**Keywords:** Regeneration, Photoreceptors, Transplantation, Retina

## Abstract

Rodent studies have recently demonstrated substantial integration of transplanted photoreceptors, with potential synaptic connection and functional restoration. Consequently, photoreceptor transplantation therapy for retinitis pigmentosa is attracting a growing interest in the field of translational research. Differentiation strategies for the formation of three-dimensional (3D) retinal tissue that are suitable for graft preparation have also been introduced via the use of human embryonic stem cells (hESCs) and human induced pluripotent stem cells. We have recently shown that hESC-derived retinal tissue (hESC-retina) can survive, mature, and potentially integrate with host secondary neurons following transplantation into two established primate models of retinal degeneration. Our data demonstrated the feasibility of deploying hESC-retina transplantation as a new remedy with which to restore the vision of patients with end-stage retinal degenerative diseases. In the present mini-review, we provide a short introduction of photoreceptor transplantation research.

## Background

**Fig. 1:**
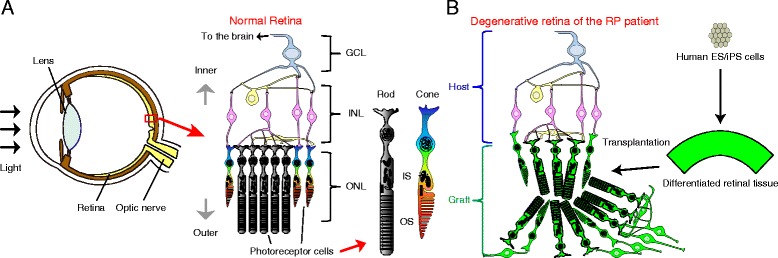
Schematic image of the human eye and therapeutic scheme of retinal transplantation. **a** Anatomical structure of the human eye and retina, which has three layers of neurons. Photoreceptor cells respond to light and are composed of rods and cones, which contain inner segments (ISs) and outer segments (OSs). **b** Retinal transplantation scheme. Retinitis pigmentosa (RP) is a group of diseases that cause progressive photoreceptor degeneration. It is hoped that retinal tissue differentiated from human ES/iPS cells could be transplanted into RP patients with a degenerative retina without photoreceptors. Although a transplanted retina contains more or less of INL components, the structured layers of photoreceptors will be in direct contact with the INL of the host retina where graft INLs had been stripped off from graft ONLs

The retina is composed of three layers of neurons, with photoreceptor cells located in the outermost layer. There are two types of photoreceptors, rods and cones, which respond to light and transfer signals to the inner neurons (Fig. 1a). Retinitis pigmentosa (RP) is a genetic disease characterized by the progressive loss of rod photoreceptors followed by secondary degeneration of cone photoreceptors, with more than 45 causal genes reported to date [1, 2]. However, only a limited number of effective treatments are currently available for this disease. Recently, photoreceptor transplantation therapy has been attracting increasing interest in the field of translational research for patients with RP (Fig. 1b). This interest stems from the first clinical trial using human retinal pigment epithelium derived from human induced pluripotent stem cells (hiPSCs), which was transplanted into the eye of a patient with advanced age-related macular degeneration [3]. Rodent studies have also demonstrated the integration of transplanted photoreceptors with potential synaptic connection and functional restoration [4–8]. Important progress has also been made in the differentiation of retinal tissue that is of an appropriate quality for grafts. Eiraku et al. developed a three-dimensional (3D) retina from mouse embryonic stem cell (ESC) aggregates using serum-free floating culture of embryoid body-like aggregates with quick reaggregation (referred to as SFEBq culture) [9]. When floating aggregates of ESCs were cultured, they spontaneously formed hemispherical vesicles that developed into the optic cup and subsequently differentiated into the outer (retinal pigment epithelium) and inner layers of the retina (neural retina). Neural retinas were then observed to grow into a stratified neural retinal tissue comprising six types of neural retinal cells. Moreover, self-organizing optic cups and 3D neural retinas have been successfully created by 3D differentiation culture from both human ESCs (hESCs) [10, 11] and hiPSCs [12], thus providing a valuable resource for grafts of any developmental stage, in any desirable form, and in quantities that are practical. In this mini-review, we briefly describe the current status of photoreceptor transplantation.

## Photoreceptor transplantation in rodent studies

Over the last decade, a number of studies in rodents have led to substantial progress being made in photoreceptor transplantation. MacLaren et al. showed that during late ontogenetic stages, photoreceptor precursors can integrate, differentiate into rod photoreceptors, form synaptic connections, and improve visual function upon transplantation into wild-type mice and mice models of early RP [4]. Subsequently, Pearson et al. demonstrated that transplanted rod-precursor cells restored vision in a murine model of congenital stationary night blindness which is devoid of rod function [6]. However, these studies were conducted with host retinas that retained the outer nuclear layer (ONL); thus, little was gained in terms of our understanding of the potential effects of transplantation in a mouse model of late-stage RP (rd1) or ONL-depleted host retinas. To solve this issue, we transplanted retinal tissue derived from murine ESCs or iPSCs into rd1 mice and subsequently demonstrated the efficacy of transplantation [8]. Transplanted retinal tissue developed a structured ONL with mature outer segments (OSs) and more or less of inner nuclear layer (INL) components. In some places, graft INL components were stripped off from structured graft layers of photoreceptors (ONL), and the uncovered photoreceptors in the grafts were revealed to be in direct 3D contact with the INL of the host retina (Fig. 1b). Detailed immunohistochemical analysis of the host-graft interface revealed a direct contact pattern indicating the successful formation of synaptic connections between graft photoreceptors and host bipolar cells, which are secondary neurons and a vital component of the INL. Collectively, these findings provide us with great hope for the future treatment of RP.

## Transplantation of hESC-derived retinal tissue into primate models of retinal degeneration

Although the efficacy of retinal tissue transplantation has been demonstrated in rodent studies, it has until recently remained unclear whether similar results can be achieved in primate models and human tissue grafts in terms of host acceptability and graft competency. We have recently evaluated this issue in a step by step manner [13]. We firstly demonstrated the ability of hESC-derived retinal tissue (hESC-retina) to survive and fully mature to form a structured ONL with inner segments (ISs) and highly specified OSs following transplantation into nude rats. Direct integration of graft photoreceptors with host bipolar cells was observed following the transplantation of graft retinas into the subretinal space of an immunodeficient rat model with end-stage retinal degeneration. We then developed two novel primate models of retinal degeneration via the subretinal injection of cobalt chloride or via 577-nm optically pumped semiconductor laser photocoagulation. We then evaluated the utility of these primate models of retinal degeneration for xenograft experiments by transplanting hESC-retina into these models. Our studies successfully demonstrated that grafts could survive and mature to form a structured ONL and potentially integrate with host bipolar cells in the eyes of our primate models. In mice, retinas derived from either ESCs or iPSCs were identical in terms of post-transplantation survival and maturation. We are currently evaluating the hiPSC-derived retina in terms of graft competency. This study provides evidence of the clinical competency of the hESC-retina and has revealed novel tools for the optimization of transplantation strategies for future clinical applications.

## Conclusions

In this mini-review, we provide a brief introduction of the current status of photoreceptor transplantation. Further research is now required to investigate whether hESC-derived retinal grafts are indeed functionally integrated. However, continued progress in this exciting discipline would finally make it possible for us to restore vision in RP patients by transplanting retinal tissue.

## Ethics approval and consent to participate

Not applicable.

## Consent for publication

Not applicable.

## Availability of data and materials

Not applicable.

## Abbreviations

3D: three-dimensional
ESCs: embryonic stem cells
hESC: human ESC
hESC-retina: hESC-derived retinal tissue
hiPSC: human induced pluripotent stem cell
INL: inner nuclear layer
IS: inner segment
ONL: outer nuclear layer
OS: outer segment
RP: retinitis pigmentosa
